# Our Unvaccinated Population Steadily Increasing

**Published:** 1921-08-20

**Authors:** 


### THE SMALLPOX QUESTION.

### Our Unvaccinated Population Steadily Increasing.

As these words are written still more cases of smallpox are being reported from the Nottingham district, and, although popular experience of the last few years seems to have blunted our perception of its potential dangers, yet it is impossible not to view, somewhat seriously, this latest outbreak. Most of us read of the first notification early in the year. Later we heard of the woman, diagnosed as chicken-pox, but actually suffering from smallpox, who travelled in a crowded railway compartment, changing carriages twice on the way. Some fifty cases have already been reported, and it is impossible to predict that there will not be more before the outbreak is finally suppressed.

From actual numbers, the incidence may not be startling, but, when we consider the active organisation employed to prevent infection, the fact that so many cases occur in spite of it should amply illustrate the danger which is threatened. Everything turns upon the much-debated question of vaccination, but it is impossible here to attempt a discussion, for, in any case, the writer's attitude is clearly displayed in this note, where only the facts of these outbreaks are quoted.

### The Instance of Nottingham.

At Nottingham, of the first fifty cases, over thirty-five have occurred in unvaccinated people. Whatever the opponents of vaccination may say, this is invariably the case in these small outbreaks. Last year, for example, Glasgow saw a vigorous outbreak ultimately negatived by a regular campaign of vaccination. At the corresponding time the precautionary measures in Edinburgh resulted in some 80,000 persons being vaccinated or re-vaccinated: there was a complete escape of Edinburgh from infection. Owing to the present law it is, however, possible for certain areas completely to refuse this protection, and, unhappily, a number of towns in the Midlands have a population refusing vaccination absolutely. To instance this *The Times* give the illustration of Coventry, which large city has steadily cast aside the greatest of all barriers against smallpox, and the table lately issued by its medical officer of health tells an obvious story.

| Year | Births | Per cent. Vaccinated |
|---|---|---|
| 1916 ... ... ... | 2,996 | 22·9 |
| 1917 ... ... ... | 2,738 | 13·0 |
| 1918 ... ... ... | 2,857 | 10·7 |
| 1919 ... ... ... | 2,429 | 8·7 |
| 1920 ... ... ... | 3,372 | 9·6 |

It does not require any deep calculation to realise how short a time it will be before such a city has a population which, to all intents, is unvaccinated. Again, we have Nottingham abundantly proving that it is mainly the unvaccinated who are attacked, and at the same time it is a recognised medical fact that the disease is vastly more serious in an unvaccinated person. Therefore, the moment of outbreak of smallpox occurs the growing numbers of unprotected people involve not only themselves, but large numbers of their fellows, in the gravest of risks.

### The Local Effect of an Outbreak.

Curiously, when the disease is actually present in a district, even a typical anti-vaccination district, there is something of a rush to obtain the protection. It is the repetition of an old story, and in the end the focus of infection will be ringed round and shut off. But when the scare dies down up will come the objections again. It is hardly too much to say that the number of unvaccinated people in this country constitutes not only a hygienic absurdity, but a disgrace to national intelligence. An objection may well be made, but for it to be legally upheld and become binding upon the local health authorities the parent should have to state and prove some very much more informed opinion than that simply ascribed to his conscience or the outpourings of cranks.

It is to the profession inconceivable that, in a country which has only just learned, in the bitter school of the war, the value of protective and preventive inoculation, and the necessity for rapidly organised, expert hygienic control in all crowded communities, there should still be allowed private "conscientious" judgment upon such a purely technical matter. As the daily Press has aptly described this public attitude, "The prejudice against inoculation was just as strong as that against vaccination. and it is exactly on the same plane as the prejudice of the people of Bombay in regard to preventive measures against plague when it is raging in their midst and slaying them by thousands." Apparently a woman in Nottingham, in utter disbelief of the commonest medical teaching, took a child suffering from acute smallpox into a crowded tram-car; while in another case an order for forcible entry had to be obtained against a woman who refused to allow the medical officer to enter an infected house! Contrast this attitude and its result with the conditions in pre-war Germany, where as might be expected perhaps, the validity of conscientious objection did not hold. Dresden had not one single case in ten years, and throughout the whole country not one special smallpox hospital was maintained.

Compulsion is a highly distasteful word to the Briton. Sometimes it seems as though certain sections of health education were equally repulsive to the British parent. On the subject of vaccination he is often hopelessly bewildered. May we call his attention to a recent paragraph published by the Research Defence Society?

### An Old Question.

Dr. Drury has provided the Society with notes of an experiment made eighteen years ago by Nature herself. There was an outbreak of smallpox in 1903 at a school in Ossett. The total number of children was 169. Of these, ninety-two were vaccinated, and seventy-seven were unvaccinated. On smallpox being introduced by one of the scholars, no less than thirty-seven of the seventy-seven unvaccinated contracted the disease. Only five of the ninety-two vaccinated contracted it. Each of these five was in a class of older scholars; and it was ten or more years since they had been vaccinated. Included among the vaccinated were fourteen who had been re-vaccinated. None of these fourteen took the disease. In Standard IV., the class in which the disease was actually introduced, some of the children were vaccinated, the rest unvaccinated. Every one of the unvaccinated took the disease, while all the vaccinated in this class escaped.

